# Disentangling direct vs indirect effects of microbiome manipulations in a habitat-forming marine holobiont

**DOI:** 10.1038/s41522-024-00503-x

**Published:** 2024-03-29

**Authors:** Alexander Harry McGrath, Kimberley Lema, Suhelen Egan, Georgina Wood, Sebastian Vadillo Gonzalez, Staffan Kjelleberg, Peter D. Steinberg, Ezequiel M. Marzinelli

**Affiliations:** 1 The University of Sydney, School of Life and Environmental Sciences, Sydney, NSW Australia; 2 Sydney Institute of Marine Science, Mosman, NSW Australia; 3 Centre for Marine Science and Innovation, School of Biological, Earth, and Environmental Science, University of New South Wales, Sydney, NSW Australia; 4 UWA Oceans Institute & School of Biological Sciences, Indian Ocean Marine Research Centre, The University of Western Australia, Sydney, Australia; 5 Singapore Centre for Environmental Life Sciences Engineering, Nanyang Technological University, 60 Nanyang Drive, SBS-01N-27, Singapore, 637551 Singapore

**Keywords:** Microbiome, Symbiosis, Biofilms

## Abstract

Host-associated microbiota are critical for eukaryotic host functioning, to the extent that hosts and their associated microbial communities are often considered “holobionts”. Most studies of holobionts have focused on descriptive approaches or have used model systems, usually in the laboratory, to understand host-microbiome interactions. To advance our understanding of host-microbiota interactions and their wider ecological impacts, we need experimental frameworks that can explore causation in non-model hosts, which often have highly diverse microbiota, and in their natural ecological setting (i.e. in the field). We used a dominant habitat-forming seaweed, *Hormosira banksii*, to explore these issues and to experimentally test host-microbiota interactions in a non-model holobiont. The experimental protocols were aimed at trying to disentangle microbially mediated effects on hosts from direct effects on hosts associated with the methods employed to manipulate host-microbiota. This was done by disrupting the microbiome, either through removal/disruption using a combination of antimicrobial treatments, or additions of specific taxa via inoculations, or a combination of thew two. The experiments were done in mesocosms and in the field. Three different antibiotic treatments were used to disrupt seaweed-associated microbiota to test whether disturbances of microbiota, particularly bacteria, would negatively affect host performance. Responses of bacteria to these disturbances were complex and differed substantially among treatments, with some antibacterial treatments having little discernible effect. However, the temporal sequence of responses antibiotic treatments, changes in bacterial diversity and subsequent decreases in host performance, strongly suggested an effect of the microbiota on host performance in some treatments, as opposed to direct effects of the antibiotics. To further test these effects, we used 16S-rRNA-gene sequencing to identify bacterial taxa that were either correlated, or uncorrelated, with poor host performance following antibiotic treatment. These were then isolated and used in inoculation experiments, independently or in combination with the previously used antibiotic treatments. Negative effects on host performance were strongest where specific microbial antimicrobials treatments were combined with inoculations of strains that were correlated with poor host performance. For these treatments, negative host effects persisted the entire experimental period (12 days), even though treatments were only applied at the beginning of the experiment. Host performance recovered in all other treatments. These experiments provide a framework for exploring causation and disentangling microbially mediated vs. direct effects on hosts for ecologically important, non-model holobionts in the field. This should allow for better predictions of how these systems will respond to, and potentially mitigate, environmental disturbances in their natural context.

## Introduction

Evidence from systems as diverse as coral reefs and the human gut indicates that host-associated microbial communities are fundamentally important for the normal development and functioning of eukaryotic hosts^1–4^. This is leading to a paradigm shift in biology, where eukaryotes are no longer viewed as single entities, but rather as part of a coherent biological association comprised of the host and their microbiome, i.e. as “holobionts”^5,6^. Most studies of holobiont ecology use observational approaches, that is, descriptions of host or microbial characteristics of the holobiont, often measured in association with variation in environmental parameters or host performance (e.g refs. ^7,8^). Such approaches typically involve sequencing and ‘omics’ techniques that have allowed detailed characterisations of microbial diversity and, in some cases, predicted function of holobionts^9–11^. Where experiments are used (see refs. ^12–14^), they are often on model organisms (although see refs. ^13–16^) in the laboratory^17–19^.

The use of model systems has enabled us to determine direct, microbially mediated effects, and their underlying mechanisms in a number of model eukaryotic hosts, particularly those associated with one or a few microbial symbionts, such as for the bobtail squid *Euphrymna scolopes* and its bacterial symbiont *Aliivibrio fisheri*^20^. Within macroalgal systems, the clearest demonstrations of the role of complex microbial communities on host function (reviewed in ref. ^21^) are from the model algae *Ulva spp*^22–24^ and *Ectocarpus spp*^12,25^. Beyond model systems, there has been work exploring the role of microbiota in the function of hosts such as mosquitos^15,26–28^ and daphnia^16,29^. However, in many of these examples of non-model hosts, the experimental manipulations occurred in the laboratory, removed from their natural environment, limiting the extent of our inferences and our capacity to predict responses of holobionts in nature.

Descriptive studies are important in providing knowledge of the diversity (who is there?) and the functional potential (what can/do they do?) of host-associated microorganisms and how microbial diversity and function vary with host performance. These studies, however, do not untangle cause-effect relationships, where the effects of complex microbial communities on hosts (and vice versa) can be distinguished and tested^30,31^. While description of patterns is a fundamental first step in the scientific framework^32^, to understand complex systems we need to move beyond descriptions to experimental manipulations that determine causation and from which potential mechanisms can subsequently be explored^33–35^. Causation indicates that one event—change in host performance - is the result of the occurrence of the other event—change in host-associated microbiota, i.e. there is a causal relationship between the two events. Importantly, not understanding the mechanisms underlying microbial effects on hosts does not impede establishing causation^36^. Relying on inferred causation only from observations (e.g. associations between variables) hinders, for example, our capacity to predict the responses of holobionts to disturbances or changing environmental conditions^31,37^. The most notable, if rare, example of an experimental system for the manipulation of complex microbiota is the use of “germ-free” mice in biomedical systems, which require intensive, whole-of-life-cycle facilities to raise mice without their normal microbiome^17,18^. While laboratory systems such as these provide important opportunities to explore direct versus indirect effects of microbial manipulation in controlled settings, their findings often do not represent the responses of natural populations^38–40^. Thus, to understand the natural ecology of, for example, key habitat forming holobionts, we need to do experiments in the field. This would align the holobiont perspective with that of macrobial ecology, when half a century ago a largely descriptive field was transformed into a strong field based experimental discipline (e.g refs. ^41–43^). Unlike in macrobial ecology, the question of how to experimentally approach holobiont ecology remains a bottleneck and has become a major focus of modern microbial ecology^31^. This is partly due to the complexity of most holobionts which harbour thousands of taxa^44–46^, giving rise to the potential for millions of interactions among these taxa and between the microbiota and the host, but is also due to the challenges of developing appropriate experimental protocols.

While significant advances on these issues have been made in model and/or laboratory systems the process of unravelling the complex associations and interactions between hosts and their microbiota is at a very early stage for most non-model holobionts living in open, “real-world” environmental systems where large, structurally complex, habitat-forming holobionts such as trees, kelps or corals create the biogenic structure of ecosystems. Understanding of host-microbiome interactions for such foundational holobionts is particularly significant because the impacts on the interaction between these hosts and their microbiota can cascade throughout entire ecosystems (e.g. ref. ^47^).

Experimental frameworks that allow establishing causation of host-microbiota interactions in holobionts are beginning to be developed^30,48^. In general, to determine microbially mediated effects of microbiota on host functioning (as opposed to direct effects on the host per se), we need experimental approaches that manipulate the microbiota in a controlled manner (where there are no effects of the manipulations other than on the microbiota) and, if possible, in a specific fashion using targeted manipulations of specific microorganisms correlated with host performance. Moreover, ideally for the environmental systems of concern here, manipulations would be conducted in the field (e.g. refs. ^49–51^) to avoid unwanted effects that are linked to the laboratory environment, as has been the case for many other biotic interactions in ecology^32^. This would have the additional advantage of integrating experiments on holobionts into the established existing framework of field experiments for macrobial ecology.

One particular challenge for such manipulations is that many of the experimental protocols do not disentangle direct effects of treatments on the hosts from effects of manipulations of the microbiota on hosts. Experimental manipulations on holobionts can involve the selective removal/reduction of microorganisms of interest (e.g., those found to correlate either positively/negatively with host performance), the addition of such microorganisms or a combination of both. The removal or reduction/disruption of host-associated microorganisms using antibiotics is a common approach (perhaps the most common approach in the field; reviewed in^17^) that has been used to study, for example, effects on host immunity, metabolism and disease^52–54^. However, the key question which arises from their use is: how do we decouple the potential direct physical/chemical effects of antimicrobials on host performance (e.g. refs. ^55,56^) from indirect, microbially-mediated effects? This is a complex and ongoing issue as different antimicrobials cause vastly different effects, both within the host and on its microbiota^57,58^.

We used a dominant, habitat-forming marine holobiont to develop and test a general experimental framework to determine direct versus indirect effects of the microbiota, i.e. cause-effect relationships between microbiota and host performance, in non-model holobionts in the laboratory and in the field. We combined microbial reduction/disruption, using antibiotics, with inoculations of specific microbial taxa associated with a widespread marine habitat-forming fucoid alga, *Hormosira banksii* (hereafter *Hormosira*). *Hormosira* forms extensive intertidal forests along Australian and New Zealand rocky shores but has declined in many urbanised areas due to multiple anthropogenic stressors (e.g. refs. ^59,60^). As with other similar seaweeds, such declines may be mediated by disruptions of their associated microbiome^8^.

We first used different antibiotic treatments that reduced/disrupted different components of *Hormosira*’s surface-associated microbiota to test the hypothesis that such microbial disruption would negatively affect host performance. To distinguish direct impacts of antibiotics from indirect, microbially-mediated effects, we isolated, and subsequently inoculated, bacterial taxa whose abundance were either correlated (and thus predicted to affect the host when added) or uncorrelated (no effect predicted) with effects on the host. By inoculating such taxa, independently or in combination with microbiota reductions over an ecologically relevant temporal scale, we were able to establish that microbial changes could affect host performance. The benefits and challenges of these approaches, individually and in concert, are explored.

## Results

### Mesocosm experiment

**Fig. 1:**
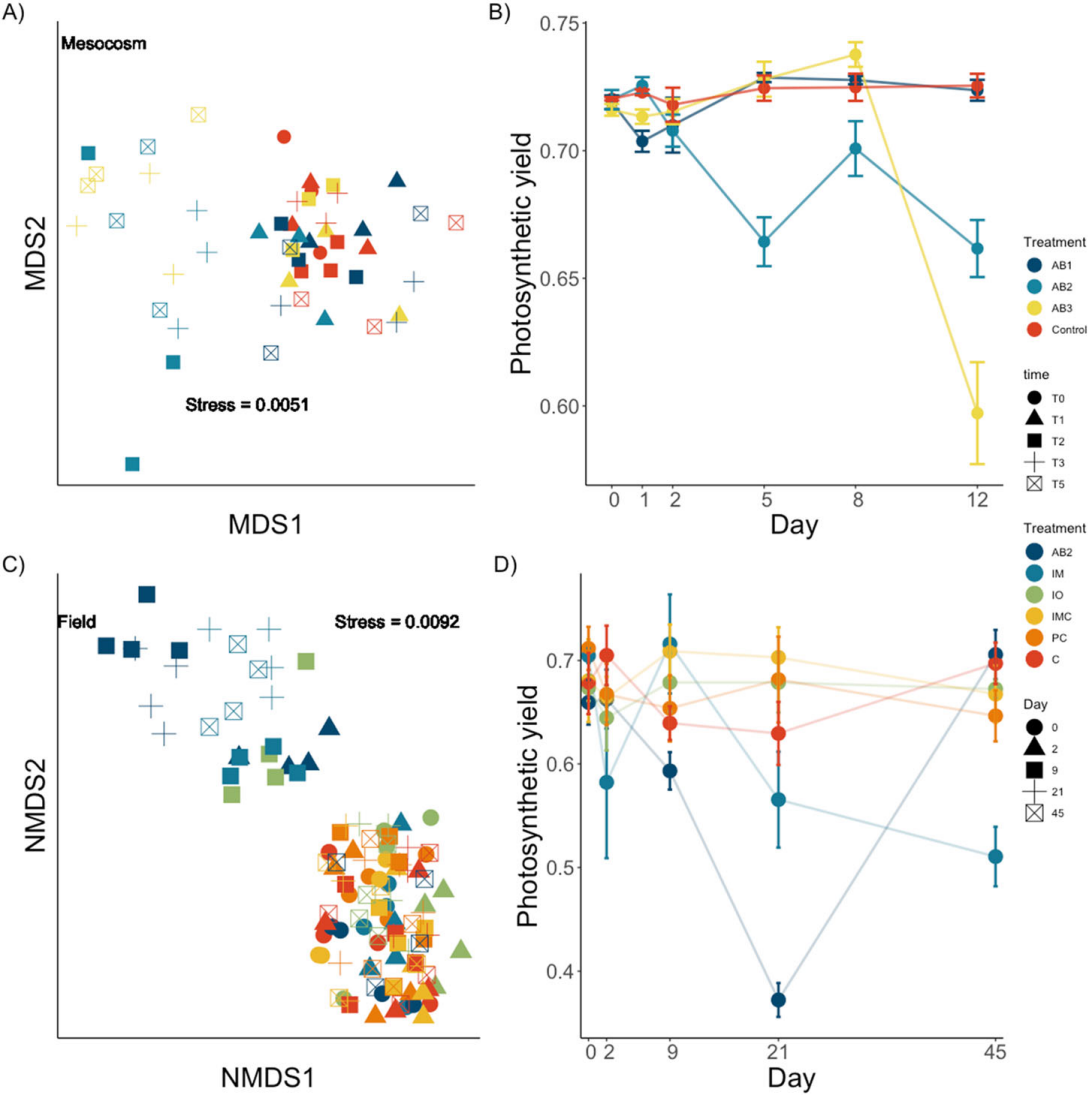
The effect of experimental manipulation on the microbial community and photosynthetic efficiency of *Hormosira banksii*. Microbial community responses Bray-Curtis dissimilarity (**A**, **C**) and, Host photosynthetic efficiency (**B**, **D**) Error bars are means +/− SE, *n* = 3; to experimental treatments in the mesocosm (**A**, **B**) and field (**C**, **D**) experiments. Microbial community data were normalised, and square root transformed prior to calculations of dissimilarities. Treatments are AB1 Antibiotic mix 1; AB2 Antibiotic mix 2, AB3 Antibiotic mix 3, C Control, IM Iodine press, IO Iodine pulse, IMC Iodine press control, PC Procedural control (AFSW).

Antibiotic treatments significantly changed the bacterial community structure on *Hormosira* in the mesocosm experiment, although, there were significant differences among different antibiotics and over time (PERMANOVA pseudo-*F*_9, 32_ = 1.29, *p* < 0.001; Supplementary Table 7; Fig. 1A). The effects of the antibiotics were not immediate, with no effect of treatments at time 1. As of day 2 *Hormosira* treated with antibiotic mix 2 had a significantly different community structure than all other treatments and that difference remained until the end of the experiment at day 12 (*F*_*9*, 32_ = 2.71, *p* < 0.001). Antibiotic mix 3’s microbial community differed significantly from controls at day 2 which lasted till day 12 (*F*_*9*, 32_ = 1.94, *p* < 0.001; Fig. 1A) and was significantly different from controls and all other treatments at day 12 (*F*_9, 32_ = 3.63, *p* < 0.001; Fig. 1A).

Community richness was significantly lower in the treatment antibiotic mix 2 than the control (ANOVA, *F*_*3, 80*_ = 6.05, *p* < 0.001; Supplementary Table 8), Antibiotic mix 3 had significantly lower richness than controls (ANOVA, *F*_*3, 80*_ = 4.84, *p* < 0.016). Simpson’s diversity index was significantly lower in the antibiotic mix 3 than all other treatments at the end of the experiment (day 12, ANOVA, *F*_*3, 80*_ = 5.49, *p* < 0.001; Supplementary Table 8).

These changes in the microbiome correlated with changes in host photosynthesis, which occurred ~3 days after treatment with AB2*. Hormosira’s* photosynthetic yield under antibiotic mix 2 experienced a continuous and significant decrease after 5 days (ANOVA, *F*_9,32_ = 8.56, *p* < *0.001*). Algae treated with antibiotic mix 3 also had decreased photosynthetic yield compared to controls, but only towards the end of the experiment, with the most significant decrease at day 12 (ANOVA, *F*_9,32_ = 7.16, *p* < *0.001*; Fig. 1B). Algae treated with the antibiotic mix 1 or in the control did not show any change in photosynthetic yield over time (Fig. 1A).

### Field experiment

In the field, bacterial relative abundance and community structure differed significantly across time and among treatments (PERMANOVA, pseudo-*F*_*20,98*_ = 1.6505, *p* < 0.001, Fig. 1C Supplementary Table 9). Antibiotics and iodine both caused significant changes in community structure from day 7 which lasted for the 46 days of the experiment (PERMANOVA, pseudo-*F*_*20,98*_ = 3.35, *p* < 0.001). Multiple applications of iodine caused greater changes in community structure than a single application alone (PERMANOVA, *F*_*5,98*_ = 3.506, *p* < 0.001). Antibiotics, when administered once, had a similar effect to multiple applications of iodine (PERMANOVA, *t* = 1.706, *p* > 0.05; Supplementary Table 9). The number of bacterial ASVs significantly decreased in algae treated with antibiotic and iodine relative to controls (ANOVA, *F*_*20,98*_ = 4.92, *p* < 0.001).

Photosynthetic yield of the host differed significantly among treatments, with individuals in the antibiotic treatment AB2 showing a significant decline in photosynthetic efficiency over time (ANOVA, *F*_*4,162*_ = 6.145, *p* < 0.001; Supplementary Table 6), starting at day 9 with the greatest effect occurring at day 21. This effect remained significant till day 46, when photosynthetic efficiency did not differ from controls (Fig. 1D; Supplementary Table 6). Algae treated multiple times with iodine (IM) also showed lower photosynthetic yield than controls and single application of iodine but did not recover by the end of the experiment. Again, this was after a lag period of 21 days (ANOVA, *F*_*20,162*_ = 3.201, *p* < 0.001; Fig. 1C; Supplementary Table 6). No other treatments differed from the controls which was coupled with no significant differences in the community composition within these treatments.

### Bacterial Isolation

To identify strains correlated with host function we isolated bacteria, leading to over 140 isolates, of which 22 strains were negatively correlated with poor host performance within both the mesocosm and field experiments. The abundance/presence of *Vibrio genomosp. F10* was most strongly correlated with poor host performance (*r* = −0.68, *p* < 0.0001; Supplementary Fig. 2) with its abundance significantly increasing in all disruption treatments except AB1 (ANOVA, *F*_*9,32*_ = 4.39, *p* < 0.001). There were also many bacterial taxa whose abundances were not correlated with host performance, e.g., *Vibrio chagasii* (*r* = 0.14, *p* < 0.12; Supplementary Fig. 2).

### Inoculation experiment

**Fig. 2:**
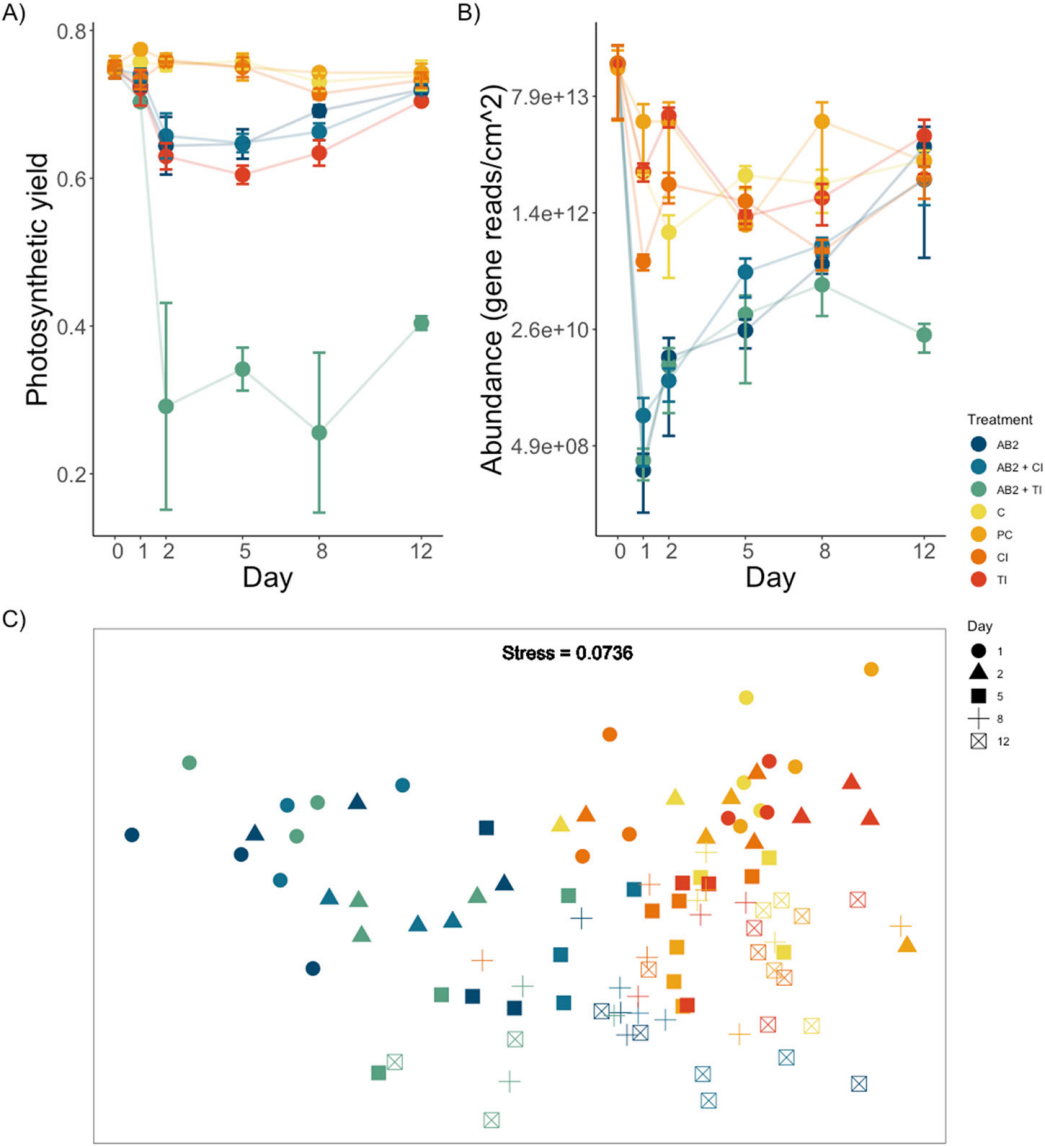
The effect of inoculation on the microbial community and photosynthetic efficiency of *Hormosira banksii*. Host and microbial community responses to experimental treatments within the inoculation experiment (Clockwise from topleft); **A** Host photosynthetic yield; **B** Absolute abundance of the 16 S rRNA gene within each treatment over time obtained with qPCR; C nMDS based on the Bray-Curtis measure on square root transformed absolute abundances of ASVs on the algal surface across treatments (stress =0.0736). Treatments are: (AB2) Antibiotic mix 2; (AB2 + CI) Antibiotic mix 2 plus control inoculant (Vibrio chagasii); (AB2 + TI) Antibiotic mix 2. error bars are means +/− SE, *n* = 3.

GLM analyses identified 172 ASVs (~1.8% of a total of 9138 ASVs) whose abundances differed significantly among treatments over the duration of the inoculation experiment. Of those, we found a strong main effect of treatment on 10 ASVs in the classes Gammaproteobacteria, Alphaproteobacteria and Cyanophyceae, with abundances of the latter two being lower in all treatments with microbiome disruption. There was a significant increase in *Vibrio* species alongside a significant increase of the inoculants *V. chagasii* and *V. genomosp. F10* whose abundances were significantly higher within inoculation treatments (ANOVA, *F*_*5,24*_ = 1.77, *p* < 0.001; Fig. 2A).

**Fig. 3:**
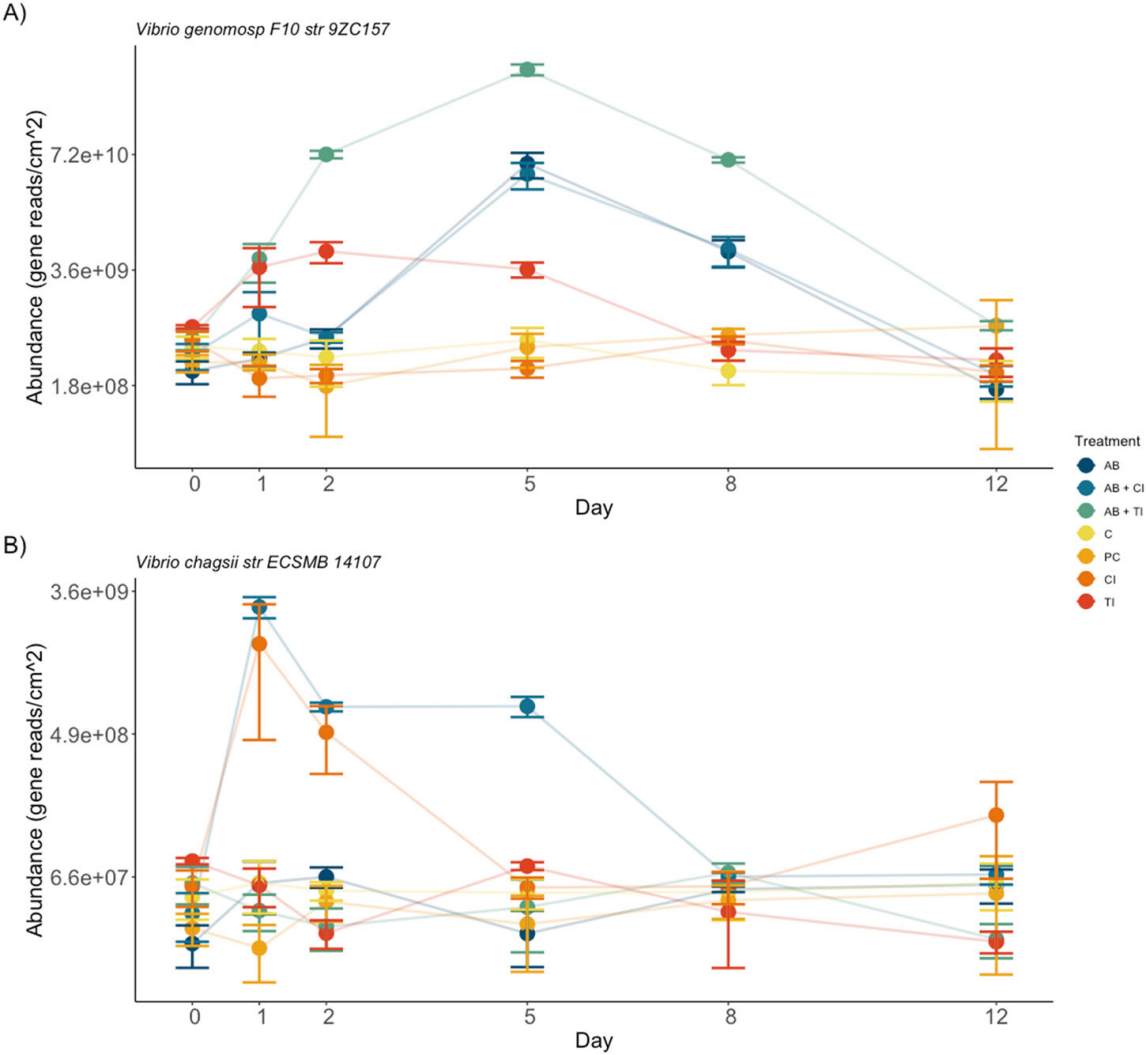
The abundances of inoculants across treatments within the inoculation experiment. Absolute abundances (error bars are means +/− SE, *n* = 3) of inoculants used within the inoculation experiment. Data are means and SE (*n* = 3) calculated using sequencing data which was normalised using qPCR of the 16 S rRNA gene; **A** Vibrio genomosp. F10; **B** Vibrio chagasii sp. across treatments and time (days). Treatments are AB2 Antibiotic mix 2, AB2 + CI Antibiotic mix 2 plus control inoculant (Vibrio chagasii), AB2 + TI Antibiotic mix 2 plus test inoculant (Vibrio genomosp. F10), C Control (Undisturbed algae), PC Procedural control (AFSW), CI Control inoculant (Vibrio chagasii), TI Test inoculant (Vibrio genomosp. F10).

Inoculation with *V. genomosp. F10*, when combined with a disruption of the microbiome, had the strongest effect on microbial community structure, which differed from all other treatments at day 2 and lasted until the end of the experiment (ANOVA, *F*_*5,24*_ = 4.28, *p* < 0.001; Fig. 3). The microbiome structure of algae disrupted with antibiotics and inoculated with *V. chagasii* did not differ from disruption with antibiotics but was different from controls (ANOVA, *F*_*5,24*_ = 1.31, *p* < 0.01; Fig. 3C) indicating that whilst the strain was present it did not influence community structure. No significant differences were found throughout the experiment among the three control treatments (Control, Procedural control, and inoculation control, Supplementary Table 12). Furthermore, all treatments with antibiotics had significantly lower richness than controls until day 8 where there was no significant difference among treatments (ANOVA, *F*_*5,24*_ = 0.969, *p* = 0.21).

All antibiotic treatments, including both treatments where the algae were further inoculated with both *Vibrio* spp., had significantly lower photosynthetic yield than those where microbiomes were left undisturbed and this effect was observed ~2 days after observed microbial changes (ANOVA, *F*_*5,24*_ = 4.25, *p* < 0.001, Fig. 3A). Inoculation with *V. genomosp. F10* had a similar effect on photosynthesis as treatments with antibiotics but without inoculants or inoculated with *V. chagasii* (ANOVA, *F*_*5,24*_ = 1.58, *p* < 0.03, Fig. 3A). All treatments, except microbial disruption followed by inoculation with *V. genomosp. F10*, recovered to the same level of photosynthetic yield that was present at the start of the experiment by day 12.

## Discussion

The structure and function of host-associated microbiomes are strongly related with host performance, but as is highlighted in the recent literature^61^, we still have very limited understanding of cause-effect in these interactions in complex, non-model holobionts within their natural ecological setting. Here, we identified the effect of the host-associated bacterial community, or subsets thereof, on the performance (as measured by photosynthetic efficiency) of a dominant, marine habitat-forming holobiont via a combination of removals and additions of microbial taxa associated with the host’s performance in mesocosms and field experiments. We disrupted the host’s microbiota using a series of different antibiotic mixes with several methods of action, but which are not thought to have direct toxic effects on multicellular algae at the concentrations used (see refs. ^62,63^). Some of these treatments had no effect on the host even though they affected the hosts microbiota providing further evidence that the observed decreases in host performance were indeed microbially mediated. We showed that the reduction/disruption of different components of the microbiota affected host performance in different ways, which changed in a non-linear fashion over time. Temporal sampling and the sequence of temporal sampling added further evidence for microbially-mediated effect being the primary consequence of these treatments. This is because any direct chemical effect of the antimicrobials on the host would likely be immediate^63,64^ (although see refs. ^65–67^) whereas microbially-mediated effects would take time following the removal/decline of taxa^68^. This was the case for our experiments in both the lab and the field. Furthermore, using inoculants which were either correlated or uncorrelated with host performance further confirmed that it was a microbially mediated effect.

In the mesocosm experiment, when exposed to antibiotics, all bacterial communities were significantly altered. These changes were followed within some treatments by decreases in host performance, i.e., there was a lag between the bacterial changes and the effect on host performance. In addition, different antibiotic mixes affected the bacterial community differently despite having similar microbial targets (Supplementary Table 2). For instance, AB1 influenced the bacterial community but not the host, while AB2 and AB3 affected the bacterial community and subsequently the host, but host responses had different lag times (3 vs 5 days after microbial changes, respectively).

These different combinations of treatments were all done to try and disentangle direct vs. Indirect (microbially mediated) effects of the treatments on the host. Because (i) bacterial changes occurred before host changes, (ii) the antibiotics had no discernible immediate effect on the host which would be expected from previous work and (iii) the three antibiotic mixes had similar MOAs but varying targets, all this suggests no (undesired) direct effects of the antibiotics on the host and changes in host performance were therefore likely caused by changes in the microbiome. These experiments and our results also speak to the challenges associated with controlling for undesired, direct effects of antimicrobial treatments on hosts. From our results, we argue that the combination of the use of several antimicrobials with similar targets but varying MOAs and chemical structures, and temporal sampling of the microbiota with host performance in parallel provides a robust approach to understanding microbially-mediated effects on host performance for non-model holobionts. The antibiotics used in our work were selected because previous studies suggested that they do not have chemical effects on the host^25^.

Other approaches for disentangling the chemical effect of antibiotics include reintroducing the microbiota in its entirety following its removal, i.e. conventionalisation^26,29^. Whilst this technique of reintroducing the microbiota has been highly successful in a number of hosts ranging from daphnia^16,29^ and mosquitos^15,26^, to zebra fish^69^ and mice^17,54^, applying this technique in the field can be highly challenging. Whilst there has been some success in hosts such as corals^51,53,70^ through the homogenisation of the hosts tissue, its applicability to other highly complex microbiota for hosts in the field is yet to be tested. Future studies could attempt this technique in the field, but should also corroborate that re-inoculating entire, complex microbial assemblages re-instates the community at similar levels of relative abundances, spatial distribution, ecological interactions and functioning of all microbial taxa (typically hundreds to thousands of ASVs) on the host as those found on unmanipulated hosts in nature.

The antibiotic mix which had the most significant and lasting effect was antibiotic mix 2 (AB2), whose difference from the other antibiotic mixes was the inclusion of the microbial RNA polymerase inhibiting Rifampicin. Antibiotic mix 3 (AB3) also contained a similar antibiotic target (Chloramphenicol) and was close to AB2’s efficacy in significantly decreasing relevant bacterial taxa from the microbiome. Indeed, the main group negatively affected by these two treatments were nitrogen-fixing cyanobacteria which we were unable to get into a pure culture. The most abundant cyanobacteria ranged from 1–3% (*Pleurocapsa* sp) of the total abundance. N-fixing bacteria such as these have been implicated in numerous symbioses with terrestrial, and more recently, marine plants^71–73^. N is a limiting nutrient within intertidal environments with many algal species often relying on extracellular N fixation to meet their physiological needs^74,75^. This may explain the observed decline in host performance.

Microbiota manipulations in the field experiment supported the results from our mesocosm experiment as microbial disruption was followed by negative effects on host performance. As in the lab, the microbial disruption caused by AB2 had a significant negative effect on host function, which eventually recovered over time (45 Days). Antiseptics (Povidone iodine 10%) had a similar, negative effect on host function when applied once (pulse) but caused lasting, negative effects on the host when applied multiple times (press). This approach mimics the natural environment closer where *Hormosira* is exposed to both press (e.g., increasing ocean temperatures^76,77^, micrograzers^78–80^) and pulse (e.g., desiccation at low tide^81^ or extreme rainfall) stressors over varying timescales.

Interestingly, cyanobacteria were the main bacteria which remained at low abundances during the press treatments both in the laboratory and in the field. Whilst our disruptions within both the lab and field show consistent patterns, many others have shown difficulty in relating findings in the lab to real-world settings (see ref. ^82^), highlighting the need to run these experiments in the field wherever possible. This is particularly important in the context of environmental change because stressors can interact in complex ways, leading to different responses of organisms depending on the environmental context^83,84^.

Disruption of the microbiota allowed for our second experimental approach—inoculations of relevant taxa – to be done in an ecologically realistic way. From the lab and field experiments we were able to isolate 22 unique strains associated with poor host performance. These isolates were dominated by readily culturable taxa from the orders Altermonadales and Vibrionales, with several ASVs being assigned to taxa that are known pathogens. Although the isolated taxa made up ~1% of total taxonomic diversity within the community, their abundance contributed to ~5% of the total. We used some of these taxa in inoculation experiments combined with microbial disruptions to determine their independent or combined effect on host performance. We were unable to culture bacterial taxa positively correlated with host function in these experiments, though we did culture taxa that were uncorrelated to performance. The inoculation of beneficial bacteria would be a logical next step for the experimental protocol developed here and could be a focus of future work on the positive effects of microbiota on host performance. Alongside this, due to the increasing recognition of the prevalence of marine fungi associated with hosts (see refs. ^85,86^), future work should also include attempts to isolate and reintroduce members of the community.

Microbial disruption, when combined with inoculations of bacterial taxa associated with poor host performance (*Vibrio genomosp. F10*), led to a much stronger negative effect on host performance than disruption or inoculation alone, and host performance did not recover during our experiment. This effect may be due to competitive release (see refs. ^87–89^), where inoculants may have been able to establish and affect the host because of the disruption/reduction of other taxa, e.g. competitors. This treatment also led to a shift in the bacterial community structure that did not recover throughout the experiment, suggesting that a threshold may have been reached where the resilience of the microbial community was overcome. Environmental stressors can lead to dysbiosis or an imbalance in the microbiota of holobionts as diverse as corals, kelps and humans, which, in turn, can make them more susceptible to being affected by pathogens^77,90^. Thus, the combination of microbial disturbance and inoculations with taxa related to poor host performance can help understand and better predict responses of holobionts to environmental stressors and the potential mechanisms underlying such responses.

Bacteria belonging to the genera *Vibrio, Pseudoalteromonas* and *Phaeobacter* have been described as antagonistic towards their hosts^91^, although this is species-specific as certain *Phaeobacter* species have been shown to provide antifouling and antilarval abilities^92^. The specificity of the effects of different strains is further demonstrated as inoculation with our control inoculant *Vibrio chagasii* spp, despite its close taxonomic relation to *Vibrio genomosp. F10*, did not induce a negative effect on host performance – this was expected as this taxon was chosen as an inoculation control because its abundance did not correlate with host performance in our first set of experiments. Sequencing analyses after inoculation showed a significant increase in the abundance of both *Vibrio chagsii* spp and *Vibrio genomosp. F10* indicating that our inoculation was successful in inducing temporary establishment within the host microbiota. However, this establishment did not last throughout the experiment; abundances of both inoculants returned to control levels by the end of the experimental period. Both strains have been reported as opportunists within other host species and are not found in high abundances within the undisturbed microbiota of *Hormosira*, which may be the result of them being outcompeted by other taxa in the microbial assemblage. This initial increase in abundance followed by rapid decrease is supported by recent work showing that commensal and mutualistic bacterial taxa switch to antagonistic pathogens when disrupted^93^.

To enable us to understand how holobionts will respond to environmental change, it is important to study them within their natural ecological settings. This is inherently difficult in natural, open systems, where thousands of taxa may contribute to millions of potential interactions. Here we have developed an experimental protocol that combines microbiota disruption using antibiotics, and the identification and subsequent inoculation of presumed harmful bacteria. This was done in both the laboratory and the field. We believe this combined approach, applied (wherever possible) in real-world settings, provides a powerful and necessary approach towards understanding of these interactions.

We showed that disruption of the host-associated microbiota using different antibiotics and subsequent temporal sampling of microbial changes and host performance provided critical information on the microorganisms potentially associated with host function, a pattern that was consistent in mesocosms and in the field. We then targeted, isolated and cultured relevant microbial taxa associated with changes in host performance (or lack thereof, as controls) and used them in controlled experiments to formally test their impact on the host. While not definitive, this approach increased the strength of our inferences as we can start to disentangle the effects of disturbance on the broader microbial community (e.g., dysbiosis caused by the antimicrobials) from the effect of individual strains of relevant microbial taxa, or both combined.

There are critical aspects that still need to be considered when interpreting outcomes of these experimental approaches, particularly the complex responses of microorganisms to different antimicrobials, the potential direct (not microbially mediated) effects on the host of the methodology employed to manipulate microbiota, and the temporal sequence of events and the environmental context (i.e. laboratory vs field). Future work should also incorporate, where possible, techniques used in laboratory experiments, such as conventionalisation, which provides a further control for manipulative experiments involving the removal/disruption of the a microbiota^17,54,69^. Another logical progression of this work is to incorporate “natural” disruptions, as those influenced by environmental changes, allowing better predictions of how these ecosystems will respond to, and potentially mitigate, future environmental disturbances.

## Methods

### Relationships between microbiome and host performance

#### Mesocosm experiment

*Hormosira* individuals of similar frond size (mean diameter: 7.8 + /− S.E 2.2 cm, length: 11.2 + /− S.E 0.81 cm; *N* = 120) were haphazardly collected by carefully detaching their holdfast from intertidal rocks during low tide at Cronulla beach, Sydney, Australia (34°03’22.8” S 151°09’19.7” E) on 4 December 2018. *Hormosira* were transported in seawater within 1 h to the flow-through aquarium facility at the Sydney Institute of Marine Science (SIMS) where they were thoroughly rinsed in autoclaved filtered (0.2 µm) seawater (AFSW) to remove fouling organisms and loosely associated microorganisms. *Hormosira* individuals were then placed into 12, 2 litre tanks (10 individuals 2.5 cm apart per tank) by cable-tying their holdfasts to weighted mesh (e.g. ref. ^94^) and left overnight to acclimatize. Tanks were supplied by filtered (5 µm), UV-treated, running seawater. The following day, algae were rinsed with AFSW and randomly assigned to one of three antibiotic combinations (AB1, AB2 or AB3; see details in Supplementary Table 2) or an AFSW control. Algae were left to soak in the different antibiotic treatments or in AFSW (control) for 24 h, then rinsed with AFSW and placed back into the 12 independent tanks (*n* = 3 tanks per treatment; 10 individuals per tank) with UV treated, 5 µm filtered seawater.

One *Hormosira* individual was randomly selected and removed from each tank prior to the addition of any antibiotics (day 0), just after their treatment with antibiotics (day 1) and every second day for 1 week and on day 12 to destructively sample individuals to characterise surface-associated microbiome and host performance (see details in Supplementary Information). Temporal sampling allows for the detection of unwanted direct effects as it is predicted that if antibiotics had any direct (e.g., chemical) effects on the host, such effects would be immediate (see ref. ^62^). On the other hand, if effects on host are microbially-mediated, it is predicted that there would be a lag between the application of the treatment and the response of the host (i.e. time for the microbiota to respond to the treatment and consequentially elicit a response on the host). Whilst temporal sampling provides evidence of direct effects if present, it is fundamentally important to also use suitable controls, i.e., in this case multiple antibiotics with similar methods of action (MOAs) but varying bacterial targets.

To characterise the associated microbiome, a consistent area of the algal surface at mid-thallus (13 + /− SE 2.7 cm^2^; older, non-meristematic tissue adjacent to, but independent from the area where photosynthesis was measured, see below) was swabbed for 30 s with sterile cotton swabs, which were immediately placed in sterile cryogenic tubes in liquid nitrogen and then stored at −80 °C until DNA extraction. After bacterial sampling, Host performance was assessed by quantifying the maximum photosynthetic quantum yield of each individual (*n* = 3, after individuals were dark-adapted for 15 min) using a Pulse Amplitude Modulated (PAM) fluorometer (WALZ, Germany), a metric of host performance widely used for photosynthetic organisms (e.g. plants and macroalgae) particularly under different environmental conditions and in relation with changes to their surface-associated microbiota^8,95–99^.

#### Field experiment

Forty-two *Hormosira* individuals of similar size (~6.8 + /− 2.3 cm in diameter and 8.5 + /− SE 1.3 cm in length) ~1 m apart were tagged at Cape Banks, Sydney, Australia (33°59’55.3” S 151°14’53.6” E) during low tide on 13 May 2018. To apply treatment to the individual alga, a sponge halo was placed over the individual alga before they were rinsed with AFSW for 1 minute and then subjected to one of 6 different treatments: (1) the antibiotic combination AB2 (chosen due to its effect on the microbiome and host performance in the mesocosm experiment above) applied once for 2 h during exposure at low tide, (2) Povidone Iodine 10% w/v (‘iodine’ hereafter) applied once for 2 h (IO), (3) iodine applied every ~2 days for 15 minutes (IM), (4) a procedural control for the treatment application once (PC; applying AFSW once for 2 h), (5) a procedural control for the continuous treatment application (IMC; applying AFSW for 15 mins every ~2 days), (6) control (C, undisturbed individual; see details in Supplementary Table 1).

Half of the surface area of each individual (~12 + /− 2.7 cm^2^) was swabbed for 30 seconds to quantify the surface-associated microbiome as described for the mesocosm experiment. Swab controls (i.e., used similarly to those for swabbing alga except no algae was swabbed) were collected. The other half was swabbed for bacterial culturing (details below); these swabs were stored in 1.5 ml Eppendorf filled with 1 ml of AFSW and placed in ice until arrival to the laboratory. Additional swab controls were collected to test for potential contamination in cultures due to the placement in AFSW. Samples were collected every 2nd day for a total of 46 days (see Supplementary Table 2 for specific dates).

#### Bacterial isolation and culture

For both the laboratory and field experiments, swabs in 1.5 ml Eppendorf tubes filled with 1 ml of AFSW were immediately taken to the laboratory for spreading on plates. Briefly, the suspended swab was vortexed and 10 ul of the solution was then plated on standard half strength marine broth agar (MP Biomedicals LLC). Individual morphologically distinct colonies were picked after 24 h. These colonies were suspended in liquid marine broth and left to grow for 48 h at room temperature on a shaker plate. 10 ul of the liquid culture was then plated as above on a new sterile agar plate. Colonies were replated until only 1 morphological form remained which was then extracted and sequenced (details below). Isolates were stored in glycerol at −80 °C until resuspension in marine broth for inoculations (see Supplementary Information for growth conditions).

Isolates were sequenced (details below) and the identity of the taxa were matched with those from the mesocosm and field experiment. Microbial amplicon sequence variant (hereafter, “ASV”) abundance was correlated with host photosynthetic yield to identify isolates for the inoculation experiments. Two strains were used for inoculations: *Vibrio genomosp (F10 str. 9*ZC157) and *Vibrio chagasii (str* ECSMB 14107). *V. genomosp F10* was chosen as its increase in abundance post-antibiotic treatment was directly correlated with poor host performance (PAM) and had the greatest change in total abundance over the experiment (Supplementary Fig. 1). *V. chagasii* was chosen as it was phylogenetically similar to *V. genomosp. F10* but remained at a constant abundance throughout the experiment and was thus uncorrelated to variation in host performance. Furthermore, the use of a control inoculation (*Vibrio* chagasii) was important to control for the potential effect of adding in a high density bacterial inoculant. For all of these reasons, *Vibrio chagasii* was therefore used as a control inoculant (Supplementary Fig. 1). In order to use both strains as inoculants they were grown to an OD of 600 before passing the cultures through a 0.2 µm filter which was being rinsed off with AFSW to the same OD.

### Testing for direct effects of components of the microbiome

#### Inoculation experiment

*Hormosira* individuals of similar length (7.5 cm +/− SE 1.2; *N* = 110) were haphazardly collected as described above from the rocky shore at Cape Banks on 25 January 2021 and transported to the SIMS aquarium within an hour, where they were rinsed with AFSW. *Hormosira* individuals (*N* = 110) were then placed into 15 ml falcon tubes which had the bottoms removed (1 individual per tube) and attached via their holdfast to a stainless-steel rod at the bottom of the tube (Supplementary Fig. 3). Each tube was individually fed filtered (50 µm 1 L/min) seawater through aquarium airlines from four replicate line splitters. Once all samples were attached in the aquarium, they were left overnight to acclimatise. On the next day, all tubes were rinsed with AFSW and the algae inside was randomly assigned to one of seven treatments (Supplementary Table 1):

1. Microbiome disruption with antibiotic combination AB2 applied once for 25 h (as in the mesocosm experiment; Supplementary Table 1);

2. Microbiome disruption as in (1) followed by inoculation with bacteria whose relative abundances correlated with poor host performance in the experiments above (*Vibrio genomosp. F10*, see Supplementary Fig. 1; cell density 5 × 10^8^ CFU);

3. Microbiome disruption as in (1) followed by inoculation with bacteria unrelated with host performance in the experiments above (negative control: *Vibrio chagsii sp*.; see Supplementary Fig. 1; cell density 5 × 10^8^ CFU);

4. Microbiome left undisturbed (AFSW) for 24 h followed by inoculation with *V. genomosp. F10* as in (2);

5. Microbiome left undisturbed (AFSW) for 24 h followed by inoculation with the negative control strain as in (3);

6. Procedural control, AFSW for 25 h;

7. Control (undisturbed algae).

Inoculations were done by immersing *Hormosira* for 1 h within the high-density cell culture which allowed sufficient time for the inoculated bacteria to attach to algal surfaces (See Supplementary Fig. 1). This timing was confirmed by leaving individuals within high cell density washed cultures for varying times before being swabbed as above. DNA was extracted and custom qPCR primers targeting the V3-V4 regions of the bacterial and archaeal 16 S rRNA gene for each of the inoculants were used to determine their abundance over time (see Supplementary Information for further details on primer design)

Samples were collected following the same timepoints as in the previous experiment: 5 individuals were collected at day 0 before treatments were applied and 3 independent individuals were randomly selected from each treatment on days 1, 2, 5, 8 and 12, at the same time of day on each sampling occasion. These time-points and the overall duration of this experiment were based on results from the previous experiments above which showed effects on microbiome and hosts within ~3–5 days (see Results) and our capacity to maintain independent replicate algae for each treatment x time combination. Surface-associated bacteria and algal photosynthetic efficiency was then characterised for each sample as described above (See mesocosm experiment above).

#### DNA extraction and sequencing

For characterisation of microbial communities in all experiments, microbial DNA was extracted from each swab sample in a randomised order using a PowerSoil DNA Isolation kit (Qiagen) following the manufacturers protocol. DNA extracts were quantified using spectrophotometry (NanoDrop 1000) and stored at -20 °C until sequencing.

The extracted DNA samples were amplified with Polymerase Chain Reaction (PCR) using the 16 S rRNA gene primers 341 (F) (5’- CCTACGGGNGGCWGCAG-3’) and 805(R) – (5’-GACTACHVGGGTATCTAATCC-‘3), covering the V3-V4 regions of the bacterial and archaeal 16 S rRNA gene^100^. The PCR conditions involved a pre-heating step to 95 °C for 3 min followed by 35 cycles of 95 °C for 15 s, 55 °C for 1 min and 73 °C for 30 s. Both positive (with known DNA sequence) and negative controls (nuclease-free water, control swabs) were used. The negative controls did not amplify DNA, suggesting no contamination on swabs/materials or during extraction and amplification. Agarose gel electrophoresis and Nanodrop 1000 were used to ensure the quantity and quality of the amplicons before they were sent for sequencing via the Illumina MiSeq 2000 platform at the Ramaciotti Centre for Genomics (UNSW, Sydney).

#### Bioinformatics

Raw sequencing data was quality filtered using Trimmomatic^101^ with a sliding window trim of 4:15 base pairs (bp) and removal of sequences with <36 bp. Paired-end reads were merged with a minimum length of 400 bp and maximum of 500 bp using USEARCH^102^. UNOISE was then used to remove chimeras and produce amplicon sequence variants (ASVs), i.e., operational taxonomic units at a unique sequence level (0% distance)^102^. USEARCH was used to map the original reads back to ASVs, generating a table of 5812, 7637 and 9138 ASVs for the mesocosm, field and inoculation experiments, respectively. ASV sequences were searched with BlastN against the SILVA SSU Ref NR99 database for taxonomic classification to classify and remove chloroplasts, the Genome Taxonomy Database (GTDB) was then used for taxonomic assignment. Singletons and low abundance taxa (<0.01% of reads) were removed from the dataset for statistical analyses, resulting in 4112, 6488 and 7028 ASVs (mesocosm, field and inoculation experiments respectively).

#### Estimation of absolute bacterial abundance

Total abundance of the 16 S rRNA gene was quantified for each sample by qPCR using the primers 341 F/805 R^103^. Gene amplification and analysis were performed using the QuantStudio 3 thermocycler (Thermo Fisher with PrimeTime® Gene Expression Master Mix, Integrated DNA Technologies) and associated software. The reaction conditions for amplification of DNA were 56 °C for 2 min, 95 °C for 10 min and 40 cycles of 95 °C for 15 s and 60 °C for 1 min. The final gene copy number per sample was corrected for the total extraction volume, the surface area and the dilution factor and DNA yield per sample (see ref. ^104^ for further details on the normalisation) and were used to estimate absolute abundances of ASVs and inoculants.

#### Statistical analyses

To account for uneven sequencing depth among samples, data were normalised using the counts per million reads method in the R package DESeq2^105^. This resulted in a total of 4,423,807, 10,443,709 and 9,921,043 sequences for the mesocosm, field experiment and inoculation datasets, respectively.

To test for effects of microbiome manipulations on host photosynthetic efficiency, we used a linear model with the factors treatment (fixed) and time (fixed, crossed). For the first mesocosm experiment where multiple *Hormosira* individuals were sampled from each tank, ‘tank’ was fitted as a random factor nested within treatment using the lme4 R package (v4.0.3). To meet the model’s assumptions, data were square root transformed. *p*-values were obtained using the anova function within the car package in R with significance being tested using F-tests, or likelihood ratio tests for the mixed model that included tank as a random effect.

Alpha diversity measures of richness (i.e., number of unique sequences) and Simpson’s diversity index were calculated using the ‘vegan’ R package^106^ and differences between treatment (fixed) and time (fixed, crossed) were examined using a linear model in the R GAD package. Where appropriate, tank was included as a random factor.

To determine differences in the structure of the associated bacterial assemblages, the normalised ASV data were analysed using permutational multivariate analysis of variance (PERMANOVA)^107^ in the R vegan package^106^, with the factors treatment, time and tank (mesocosm experiment only) as above. Similarity matrices were calculated using Bray-Curtis measure on square-root transformed data and visualised through non-metric multi-dimensional scaling (nMDS) ordinations. We also calculated and plotted mean Bray-Curtis similarities to the control (undisturbed algae) treatment. To determine which bacterial taxa’s abundance differed among treatments and times, we used multivariate generalised linear models (GLMs) using the R package ‘mvabund’^108^, assuming a negative-binomial distribution to account for over-dispersion. To determine how the total abundance of the two inoculants changed over the course of the experiment, linear models were fitted with the factors treatment (fixed) and time (fixed, crossed) in R (v4.0.3).

### Reporting summary

Further information on research design is available in the Nature Research Reporting Summary linked to this article.

### Supplementary information

Supplementary information: Disentangling direct vs indirect effects of microbiome manipulations in a habitat-forming marine holobiont

Reporting Summary

## Data Availability

All data, sampling locations, amplicon sequence data, host response and timepoints are available on zenodo 10.5281/zenodo.8031031.
