# Publisher Correction: Disentangling direct vs indirect effects of microbiome manipulations in a habitat-forming marine holobiont

**DOI:** 10.1038/s41522-024-00515-7

**Published:** 2024-05-03

**Authors:** Alexander Harry McGrath, Kimberley Lema, Suhelen Egan, Georgina Wood, Sebastian Vadillo Gonzalez, Staffan Kjelleberg, Peter D. Steinberg, Ezequiel M. Marzinelli

**Affiliations:** 1 The University of Sydney, School of Life and Environmental Sciences, Sydney, NSW Australia; 2 Sydney Institute of Marine Science, Mosman, NSW Australia; 3 Centre for Marine Science and Innovation, School of Biological, Earth, and Environmental Science, University of New South Wales, Sydney, NSW Australia; 4 UWA Oceans Institute & School of Biological Sciences, Indian Ocean Marine Research Centre, The University of Western Australia, Sydney, Australia; 5 Singapore Centre for Environmental Life Sciences Engineering, Nanyang Technological University, 60 Nanyang Drive, SBS-01N-27, Singapore, 637551 Singapore

**Keywords:** Microbiome, Symbiosis, Biofilms

Correction to: *npj Biofilms and Microbiomes* 10.1038/s41522-024-00503-x, published online 29 March 2024

In this article the legend/color bars for Figs.1–3 were inadvertently omitted from the figure. The original article has been corrected.

