# Supplementary material for: Disentangling direct vs indirect effects of microbiome manipulations in a habitat-forming marine holobiont
Source: NPJ Biofilms Microbiomes. 2024 Mar 29;10:33. doi: 10.1038/s41522-024-00503-x (PMC10980776; doi:10.1038/s41522-024-00503-x)
Supplement: Supplementary file 1 — Supplementary information: Disentangling direct vs indirect effects of microbiome manipulations in a habitat-forming marine holobiont [file 41522_2024_503_MOESM1_ESM.pdf]

Supplementary information: Disentangling direct vs indirect effects of microbiome manipulations in a habitat-forming marine holobiont

Alexander H. McGrath[1,2,*], Kimberley Lema[2,3], Suhelen Egan[2,3], Georgina Wood[1,2,5], Sebastian Vadillo Gonzalez[1, 2], Staffan Kjelleberg[4], Peter D. Steinberg[2,3,4], Ezequiel M. Marzinelli[1,2,4]

[1]The University of Sydney, School of Life and Environmental Sciences, Sydney, New South Wales, Australia

[2] Sydney Institute of Marine Science, Mosman, New South Wales, Australia

[3] Centre for Marine Science and Innovation, School of Biological, Earth, and Environmental Science, University of New South Wales, Sydney, New South Wales, Australia

[4] Singapore Centre for Environmental Life Sciences Engineering, Nanyang Technological University, 60 Nanyang Drive, SBS-01N-27, Singapore 637551, Republic of Singapore

[5] UWA Oceans Institute & School of Biological Sciences, Indian Ocean Marine Research Centre, The University of Western Australia

*Corresponding author. Email: alexander.mcgrath@sydney.edu.au

Short title: Causation in host-microbiome interactions

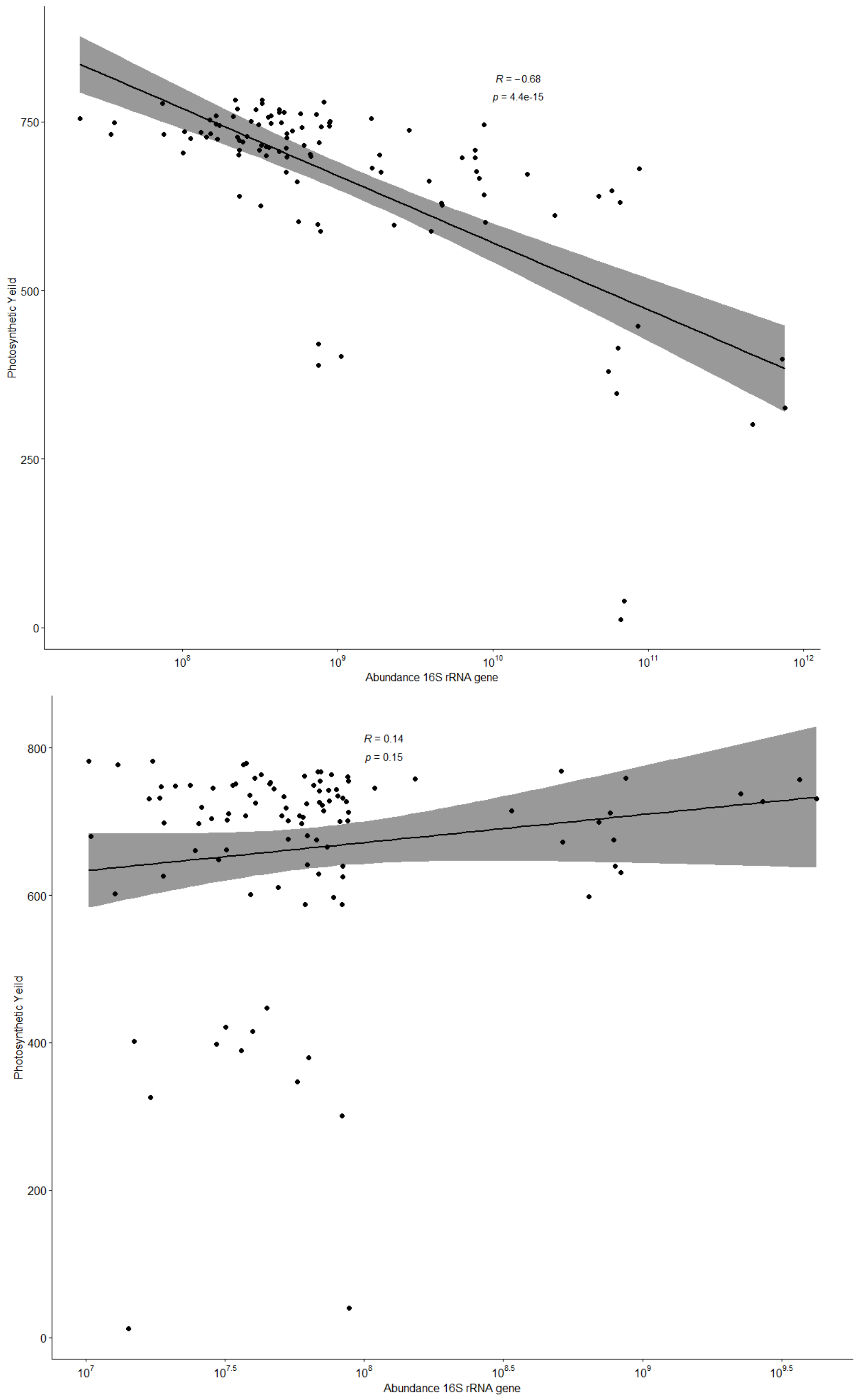

Supplementary figure 1 – Correlation plots between isolates (a) *Vibrio genomosp sp* and (b) *Vibrio chagasii* and host function (Photosynthetic yield)

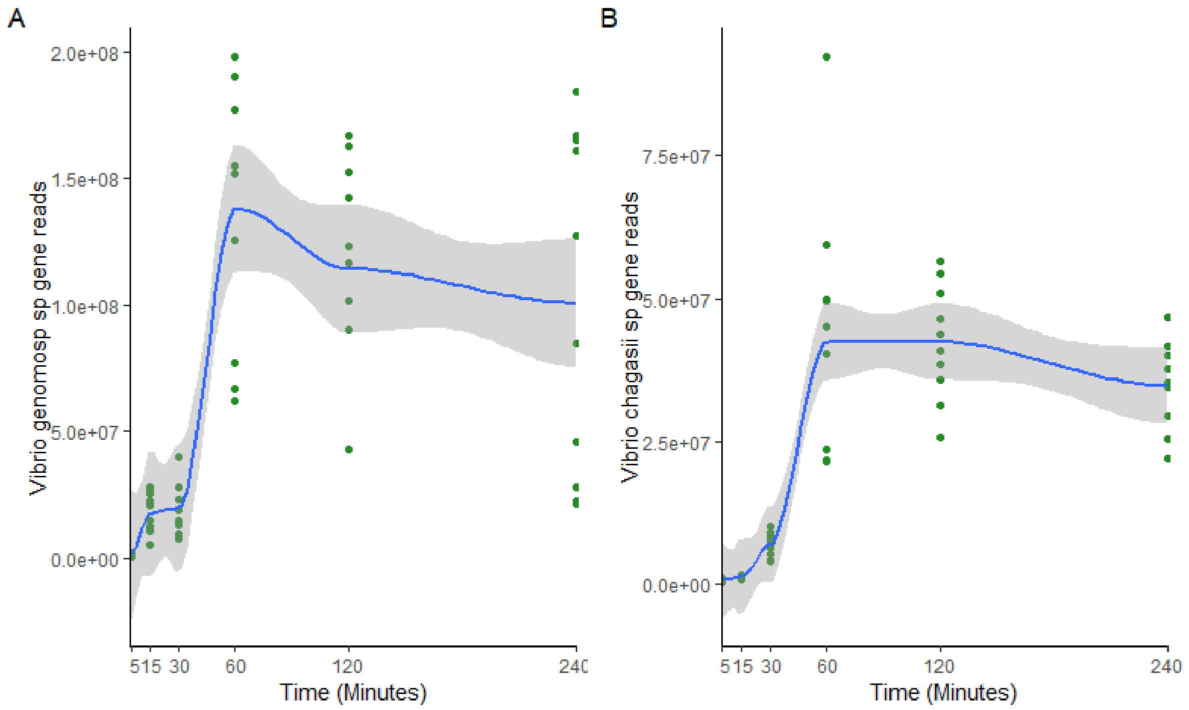

Supplementary figure 2 – Abundances of gene reads extracted from qPCR data using custom primers (A) *Vibrio genomosp sp* and (B) *Vibrio chagasii*.

.

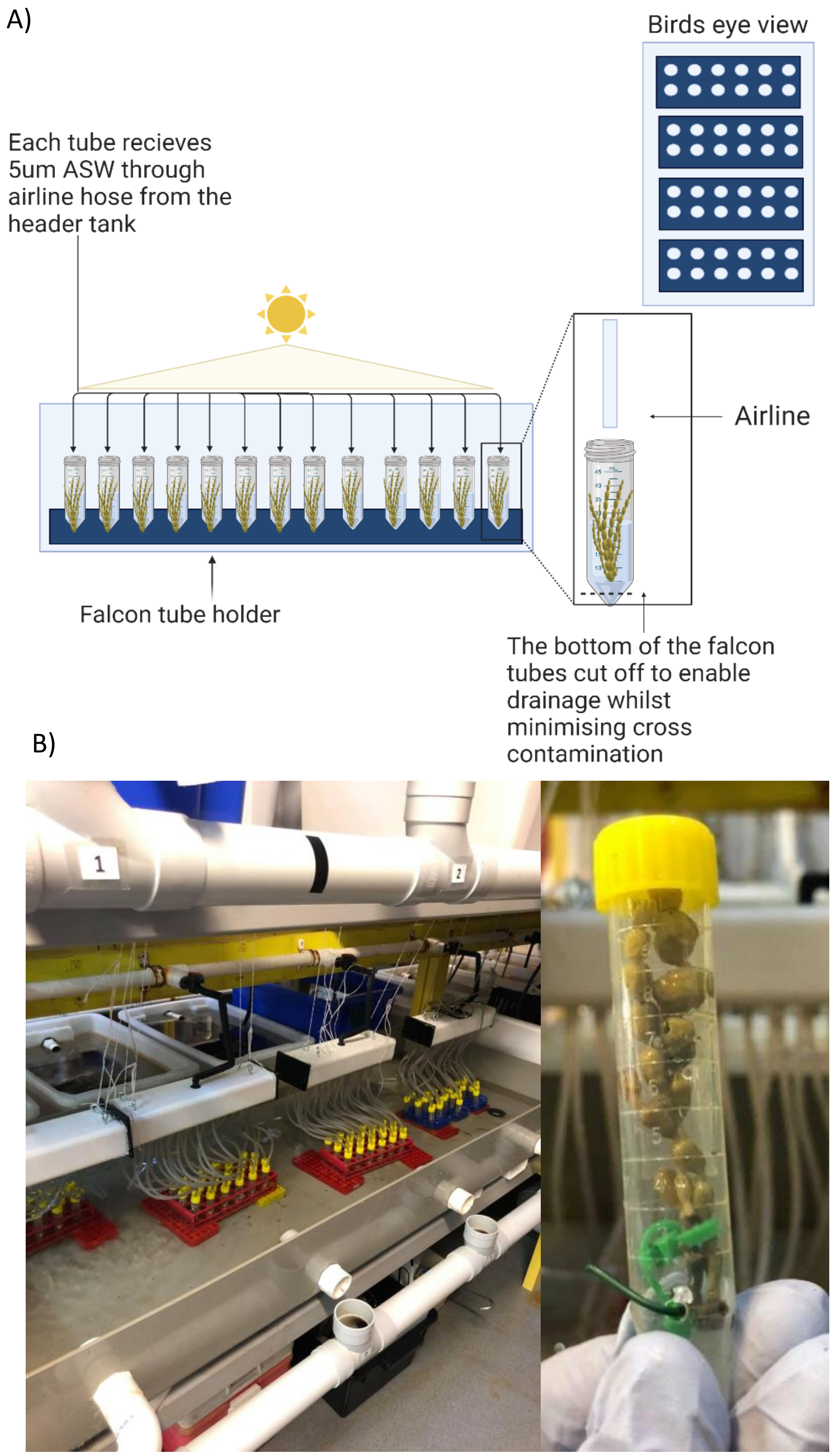

Supplementary figure 3 – Experimental setup showing schematic diagram (A) alongside actual setup (B). Each individual algal was attached by their holdfast to the bottom of a 15ml falcon tube. Each tube is then fed an individual and constant supply of 5um filtered seawater

Supplementary Table 1; Description of treatments used within the 3 experiments (Mesocosm, Field and Inoculation)					
**Experiment**	**a) Abbreviation**	**b) Treatment's specification**	**c) Treatment application**	**d) Sampling times for amplicon sequencing**	**e) Sampling times for Photosynthetic yield**
**Mesocosm experiment**	AB1	Antiobiotic treatement 1: Penincilin (100mg/ml)/Streptomycin(25mg/ml)/Norfloxacin (1mg/ml)/Kanamycin (25mg/ml)	24 hours in antibiotic solution and rinsed with AFSW	Day 1 (24hours after antibiotic treatement-06/12/2018); day 2 (7/12/2018); day 5(10/12/2018); day 12 (17/12/2018)	Day 0 (before antibiotic treatment-05/12/2018); day 1 (24hours after antibiotic treatement-06/12/2018); day 2 (7/12/2018); day 5 (10/12/2018); day 7 (12/12/2018); day 12 (17/12/2018); day 16(21
	AB2	Antiobiotic treatement 2: Penincilin (100mg/ml)/Neomycin(100mg/ml)/Rifampicin(50mg/ml)	24 hours in antibiotic solution and rinsed with AFSW		
	AB3	Antiobiotic treatement 3: Penincilin (100mg/ml)/Streptomycin(25mg/ml)/Norfloxacin (1mg/ml)/Kanamycin (25mg/ml)/ Neomycin(100mg/ml)/ Chloramphenicol (10mg/ml)	24 hours in antibiotic solution and rinsed with AFSW		
	Control	Control (no antibiotics)	24 hours in AFSW and rinsed with AFSW at the same time then rinsing of antibiotic treatements		

**Field experiment**	AB2	Antibiotic treatement 2 (as in laboratory experiment but higher concentrations): Penincilin (900mg/ml)/Neomycin(900mg/ml)/Rifampicin(600mg/ml)	Quick AFSW rinse and application of AB2 for 2 hours and thorough rinse with AFSW	Day 0 (before applying any treatment- 13/05/19); day 2 (2 days after treatment application- 15/05/19); day 9 (22/05/19); day 21 (03/06/19); day 45 (27/06/19)	Day 0 (before applying any treatment- 13/05/19); day 2 (2 days after treatment application- 15/05/19); day 4 (17/05/19); day 7 (20/05/19); day 9 (22/05/19); day 14 (27/05/19); day 16 (29/05/19); day 21 (03/06/19); day 28 (10/06/19); day 36 (18/06/19); day 40 (22/06/19); day 45 (27/06/19)
	BetO	Betadine applied once (Povidone iodine solution 10%)	Quick AFSW rinse and application of Betadine for 2 hours and thorough rinse with AFSW		
	BetM	Betadine applied multiple times (Povidone iodine solution 10%)	Quick AFSW rinse and application of Betadine for 15 minutes and rinsed with AFSW. Same process performed approx. every 2 days (same days then photosynthetic yield sampling see column e)		
	ProcContO	Procedural control for AB and CBetO	Quick AFSW rinse and application of		

			AFSW for 2 hours and thorough rinse with AFSW		
	ProcContM	Procedural control for BetM	Quick AFSW rinse and application of AFSW for 15 minutes and rinsed with AFSW. Same process performed approx. every 2 days (same days then BetM and photosynthetic yield sampling see column e)		
	Control	General control	Quick AFSW rinse		
**Inoculation experiment**	AB2	Antibiotic treatment 2: Penincilin (100mg/ml)/Neomycin(100mg/ml)/Rifampicin(50mg/ml)	Quick AFSW rinse and application of AB2 for 25 hours and thorough rinse with AFSW	Day 0 (Before treatment), Day 1, Day 2, Day 5, Day 8, Day 12	

	AB2 V+	Antibiotic treatment with Active inoculant	microbiome disruption with antibiotic combination (AB2) for 24 hours followed by “active” inoculant (*Vibrio genomosp*, Cell density 5×10^8 CFU) for 1 hour,	
	AB2 V-	Antibiotic treatment with Control inoculant	microbiome disruption with antibiotic combination (AB2) for 24 hours followed by control inoculant (*Vibrio chagsii sp.* Cell density 5×10^8 CFU) for 1 hour,	
	Vibrio +	Active inoculant	microbiome left intact (AFSW) for 24 hours followed by “active” inoculant (*Vibrio genomosp*,Cell density 5×10^8 CFU) for 1 hour	

	Vibrio -	Control Inoculant	microbiome left intact (AFSW) for 24 hours followed by control inoculant (*Vibrio chagsii sp.* Cell density 5×10^{8} CFU) for 1 hour	
	PC	Procedural control	Algae left in AFSW for 25Hrs	
	C	Control	Algae left in aquarium during treatment	

Supplementary Table 2 Method of action and key bacterial targets of antibiotics used

**Antibiotic**	**Method of Action**	**Bacterial target**	**References**	**Antibiotic mix**
Penincilin	Inhibition of transpeptidase - inhibition of cell wall biosynthesis	Gram-positive bacteria	Tipper and Trominger, 1965; Waxman et al 1980; Sass, 2023	1,2,3
Streptomycin	Interference with ribosomal peptide synthesis	Limited aerobic bacteria due to antibiotic resistance	Demirci et al 2013	1, 3

Norfloxacin	Inhibits DNA gyrase	Most gram-negative and gram-positive bacteria	Goldstein 1987	1, 3
Kanamycin	Binding to bacterial 30S and 16S ribosomal subunit – causes misreading of t-RNA	Gram-negative bacteria	Brezden et al 2016	1, 3
Neomycin	Binding to bacterial 30S ribosomal subunit – inhibits elongation of peptide synthesis	Gram-negative bacteria	Veirup et al 2022; Patidar et al 2013	2
Rifampicin	Inhibits DNA-dependant RNA-polymerase	Gram-positive cocci	Campbell et al 2001; McClure et al 1978	2
Chloramphenicol	Bacteriostatic inhibitor of protein synthesis – binds to bacterial 50S ribosomal subunit	Gram-positive and gram-negative bacteria	Brook et al 2016; Dinos et al 2016	3

Supplementary Table 3. Statistical results. Analysis of Variance (ANOVA) tests comparing maximum photosynthetic yields of *Hormosira banksii* across sampling time points and different treatments for the mesocosm experiment

**Source**	**Df**	**Sum of Sq**	**Mean Sq**	**F**	**P-Value**
**Treatment**	3	0.041843	0.0139476	20.645	5.30E-10
**Sampling time**	3	0.42831	0.0142768	21.133	3.54E-10

**Treatment*Sampling time**	9	0.11584	0.0128712	19.052	<2.2E-16
**Residual**	80	0.054047	0.0006756		

Supplementary Table 4; Post-hoc analysis comparing maximum photosynthetic yields of *Hormosira banksii* across sampling time points and different treatments for the mesocosm experiment

**Days**	**Treatment comparison**	**estimate**	**SE**	**df**	**t.ratio**	**p.value**
**0d**	**No Significant differences**					
**1d**						
**2d**						
**5d**	AB2-AB1	0.085644	0.015	80	5.707	<.0001
	AB3-AB2	0.082365	0.015	80	5.489	<.0001
	Control-AB2	0.088333	0.015	80	-5.886	<.0001
**9d**						
**12d**	AB2-AB1	0.085644	0.015	80	5.707	<.0001

	AB3-AB1	0.168009	0.015	80	11.196	<.0001
	AB3-AB2	0.082365	0.015	80	5.489	<.0001
	Control-AB2	-0.088333	0.015	80	-5.886	<.0001
	Control-AB3	-0.170698	0.015	80	-11.375	<.0001

Supplementary Table 4; Statistical results. Analysis of Variance (ANOVA) tests comparing maximum photosynthetic yields of *Hormosira banksii* across sampling time points and different treatments for the field experiment

**Source**	**Df**	**Sum of Sq**	**Mean Sq**	**F**	**P-Value**
**Treatment**	5	0.21121	0.042241	5.6901	***7.30E-05***
**Sampling time**	4	0.15841	0.039604	5.3348	***4.63E-04***
**Treatment*Sampling time**	20	0.65118	0.032559	4.3859	***4.29E-08***
**Residual**	162	1.20263	0.007424		

Supplementary Table 5; Post-hoc analysis comparing maximum photosynthetic yields of *Hormosira banksii* across sampling time points and different treatments for the field experiment						
**Days**	**Treatment comparison**	**estimate**	**SE**	**df**	**t.ratio**	**p.value**
**0d**	**No Significant differences**					
**1d**						
**2d**						
**5d**						
**9d**	**Control-AB**	**-0.15014**	**0.0461**	**162**	**-3.260**	**0.0168**
	**ProcContM-BetM**	**0.19557**	**0.0461**	**162**	**4.247**	**0.0005**
	**ProcContM-BetO**	**0.21157**	**0.0461**	**162**	**4.594**	**0.0001**
**12d**						
**21d**	**BetM-AB**	**-0.18786**	**0.0461**	**162**	**-4.079**	**0.0010**
	**BetO-AB**	**-0.26243**	**0.0461**	**162**	**-5.698**	**<.0001**

	**ProcContM-AB**	**-0.25743**	**0.0461**	**162**	**-5.590**	**<.0001**
	**ProcContO-AB**	**-0.24914**	**0.0461**	**162**	**-5.410**	**<.0001**
	**Control-AB**	**-0.28300**	**0.0461**	**162**	**-6.145**	**<.0001**
**45d**	**BetM-AB**	**0.19525**	**0.0609**	**162**	**3.205**	**0.0199**
	**Control-BetM**	**-0.186750**	**0.0583**	**378**	**-3.201**	**0.0185**

Supplementary Table 6 Statistical results. ANOVA results for analysis of bacterial community alpha diversity measures: number of species and Simpson index in Hormosira banksii individuals within the mesocosm experiment.

**Source**		**Df**	**Sum of Sq**	**Mean Sq**	**F**	**P-value**
Treatment		3	73457	24486	6.0464	0.00221
Sampling time		6	132847	44282	10.9348	4.22e-05
Treatment x Sampling time		9	38336	4260	1.0518	0.42293
Residual		32	129589	4050		

Table S7. Statistical results. ANOVA results for analysis of bacterial community alpha diversity measures: number of species and Simpson index in Hormosira banksii subject to different treatments across time in the field). Post-hoc pairwise comparisons are also presented (only showing results of time points and treatments with significant differences).

**Source**	**effect size**	**Df**	**Sum of Sq**	**Mean Sq**	**F**	**P-value**
Treatment	0.15	5	101780	20356	4.9231	***0.0004984***

Sampling time	0.21	4	143095	35774	8.6519	***5.911e-06***
Treatment x Sampling time		20	69236	3462	0.8372	0.6631976
Residual		98	372129	4135		
**Pairwise test**						
Treatment	AB2 - Control	p<0.005**				
	AB2 - CBetM	p<0.5*				
	AB2 -CBetO	p<0.5*				
	BetM - Control	p<0.5*				
	BetM- CBetO	p<0.5*				

Supplementary Table 8; PERMANOVAs based on Bray–Curtis Similarity measure for square root-transformed relative abundance (9999 permutations) of bacterial communities (Zotus) in Hormosira banksii across sampling time points and different treatments the mesocosm experiment. Table S1 has the treatments used. Significant p values(p<0.05) are highlighted in bold and italics.

Source	df	SS	MS	Pseudo-F	P(perm)	perms
Treatment	3	5699.3	1899.8	2.1882	**0.0001**	9798
Sampling time	3	19463	6487.7	7.4726	**0.0001**	9884
Treatment x Sampling time	9	10151	1127.9	1.2991	**0.0014**	9677
Tank	6	1059	176.5	1.862	0.3298	9786
Residual	32	27782	868.19			
Total	47	63095				

**Supplementary Table 9; Pairwise contrasts on PERMANOVAs based on Bray–Curtis Similarity measure for square root-transformed relative abundance (9999 permutations) of bacterial communities (Zotus) in Hormosira banksii across sampling time points and different treatments the mesocosm experiment.**			
**Pairwise test**	**Days**	**Treatment comparison**	
Treatment x Sampling time	12d	AB2 - AB1	p<0.005**
		AB3 - Control	p<0.5*
		AB3 - AB1	p<0.005**
Treatment	AB2 - Control	p<0.005**	
	AB2 - AB1	p<0.005**	
	AB2 - AB3	p<0.5*	

Supplementary Table 10. PERMANOVAs based on Bray–Curtis Similarity measure for square root-transformed relative abundance (9999 permutations) of bacterial communities (Zotus) in Hormosira banksii across sampling time points and different treatments the field experiment. Table S1 has the treatments used. Significant p values(p<0.05) are highlighted in bold and italics.

Source	**df**	**SS**	**MS**	**Pseudo-F**	**P(perm)**	**perms**
Treatment	5	22032	4406.4	7.6284	***0.0001***	9811
Sampling time	4	33213	8303.2	14.374	***0.0001***	9846
Treatment x Sampling time	20	26297	1314.9	2.2763	***0.0001***	9591
Residual	90	51987	577.64			

Total	119	133530				

Supplementary Table 11. PERMANOVAs based on Bray–Curtis Similarity measure for square root-transformed relative abundance (9999 permutations) of bacterial communities (Zotus) in Hormosira banksii across sampling time points and different treatments the field experiment. Table S1 has the treatments used. Significant p values(p<0.05) are highlighted in bold and italics.

**Days**	**Treatement comparison**	**t**	**P(perm)**	**perms**	**P(MC)**	
**0d**	**No significant differences**					
**2d**	**BetO- ProcContO**	2.3292	0.0292	35	***0.0076***	
	**BetO- ProcContM**	2.265	0.0292	35	***0.0091***	
	**BetO- Control**	2.1701	0.0247	35	***0.0135***	
	**ProcContO- BetM**	2.8772	0.0265	35	***0.0033***	
	**ProcContO- AB**	1.9269	0.0289	35	***0.0142***	
	**BetM- ProcContM**	2.6858	0.0294	35	***0.0033***	
	**BetM- Control**	2.446	0.03	35	***0.0068***	
	**BetM- AB**	1.7065	0.0279	35	***0.0403***	
	**ProcContM- AB**	1.8055	0.0308	35	***0.0188***	
	**Control- AB**	1.5982	0.0298	35	***0.0419***	
**9d**	**BetO- BetM**	2.7522	0.0319	35	***0.0026***	
	**BetO- Control**	1.7677	0.0292	**35**	***0.026***	
	**BetO- AB**	1.7588	0.0296	35	***0.0366***	
	**ProcContO- BetM**	3.2668	0.0287	35	***0.0012***	

	**ProcContO- AB**	1.8704	0.0303	35	***0.0195***	
	**BetM- ProcContM**	3.5876	0.0312	35	***0.0007***	
	**BetM- Control**	3.4544	0.0275	**35**	***0.0008***	
	**BetM- AB**	2.589	0.0313	35	***0.0049***	
	**ProcContM- AB**	2.013	0.0273	35	***0.0152***	
	**Control- AB**	1.8592	0.0256	**35**	***0.0198***	
**21d**	**BetO- BetM**	3.0037	0.0292	35	***0.0024***	
	**ProcContO- BetM**	3.5358	0.0313	35	***0.0009***	
	**ProcContO- AB**	1.6949	0.0285	35	***0.0299***	
	**BetM- ProcContM**	4.3121	0.0281	35	***0.0008***	
	**BetM- Control**	3.2017	0.0285	**35**	***0.0013***	
	**BetM- AB**	2.7522	0.03	35	***0.0019***	
	**ProcContM- AB**	2.016	0.0287	35	***0.0104***	
	**Control- AB**	1.5638	0.0267	**35**	***0.0501***	
**45d**	**BetO- BetM**	2.2648	0.0277	35	***0.0061***	
	**ProcContO- BetM**	2.7101	0.0285	35	***0.0031***	
	**BetM- ProcContM**	2.4161	0.0261	35	***0.0037***	
	**BetM- Control**	2.6674	0.0292	**35**	***0.003***	
	**BetM- AB**	2.5228	0.0286	35	***0.004***	
